# Adhesion force mapping on wood by atomic force microscopy: influence of surface roughness and tip geometry

**DOI:** 10.1098/rsos.160248

**Published:** 2016-10-19

**Authors:** X. Jin, B. Kasal

**Affiliations:** 1 Organische Baustoffe und Holzwerkstoffe, TU Braunschweig, Hopfengarten 20, 38102 Braunschweig, Germany; 2 Fraunhofer Wilhelm-Klauditz-Institut WKI, Bienroder Weg 54E, 38108 Braunschweig, Germany

**Keywords:** atomic force microscopy, wood, natural fibre, surface roughness, adhesion force measurement, tip geometry

## Abstract

This study attempts to address the interpretation of atomic force microscopy (AFM) adhesion force measurements conducted on the heterogeneous rough surface of wood and natural fibre materials. The influences of wood surface roughness, tip geometry and wear on the adhesion force distribution are examined by cyclic measurements conducted on wood surface under dry inert conditions. It was found that both the variation of tip and surface roughness of wood can widen the distribution of adhesion forces, which are essential for data interpretation. When a common Si AFM tip with nanometre size is used, the influence of tip wear can be significant. Therefore, control experiments should take the sequence of measurements into consideration, e.g. repeated experiments with used tip. In comparison, colloidal tips provide highly reproducible results. Similar average values but different distributions are shown for the adhesion measured on two major components of wood surface (cell wall and lumen). Evidence supports the hypothesis that the difference of the adhesion force distribution on these two locations was mainly induced by their surface roughness.

## 1. Introduction

Atomic force microscopy (AFM) has been a useful method in adhesion force measurements, owing to its ability to identify surface forces at precise locations, and its suitability for various materials and environmental conditions. Experiments and theories are relatively well developed for measurements on homogeneous and atomically smooth surfaces [1]. Attempts have also been made to study the surface of wood and natural fibre materials in order to gain localized surface energy information in the submicron structures [2–4]. These materials represent considerable challenge in using the AFM method owing to their inherent variability and their natural surface pattern, which often has roughness in multiple scales. Studies have shown that on rough surfaces, the AFM adhesion measurements are generally challenging and require special attention [5–8]. Measurements conducted under ambient conditions are also known to be problematic for interpretation [1,9,10]. These issues are particularly pronounced in the case of wood and natural fibres. First of all, the hydrophilic materials absorb various amount of water under various humidity levels [11]. Furthermore, their natural surfaces have distinct textures and thus various roughness that can result in more complex wetting behaviour compared with smooth surfaces. This study attempts to address the reproducibility and interpretation of adhesion measurements with AFM on natural fibre surfaces. Repeated experiments were carried out on surfaces of coniferous wood under inert conditions. The influence of various surface roughness of wood cell components, with respect to the geometry of common commercially available tips, is analysed. The reproducibility and effect of tip wear are also discussed. The results could provide valuable information for the use of AFM adhesion force measurements on wood and other heterogeneous rough materials.

Adhesion as a scientific term has been used under various contexts [12]. In this paper, we use the term ‘adhesion’ or ‘adhesion measured’ in the sense of the force against separation measured by the difference between approach and retraction curves in an AFM force measurement. The practical adhesion obtained here, like in any other mechanical tests, is influenced by the mechanical parameters such as preload, loading rate, etc. Instead of the absolute values, which reflects on the mechanical response and rheological properties of the system [13], we are more interested in the relative values which provide information about the fundamental adhesion. Adhesion between two objects fundamentally depends on the surface chemical properties and the area in contact. In the case of AFM measurement on a rough surface, it is critical to know the exact surface topography involved in the adhesion measurements. These include the substrate topography, and the AFM tip topography/geometry. For complex surfaces, the key for successful predication of the adhesion force lies on the precise modelling of surface topography. Existing theories use roughness, asperity height and radius, etc. as parameters, or fractals and Fourier transformation algorithm to describe the surface [1,14]. Despite their usefulness, these models are mostly based on contact measurements on hard substrates. The elasticity and deformation of a rough surface in contact is considerably complex, which still needs to be studied in detail.

**Figure 1.**
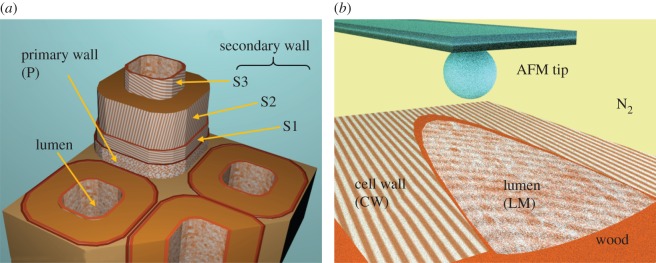
(*a*) Illustration of wood cell structure (redrawn from Haygreen & Bowyer [11]). The fibre orientation differs between layers. The S2 and S3 layers compose the most wood surface, which were cut along primary growth direction. (*b*) Experimental set-up: adhesion force was measured by pulling off an AFM tip from the selected location on a wood cell in a nitrogen atmosphere. The difference between approach and retract curves is used to calculate the adhesion force.

Owing to its cellular structure, the cut surface of wood consists of diverse components and hollow spaces, as shown in figure 1*a*. We examine here representatively the two major surface parts in the case of a softwood: the secondary cell wall (mostly S2) and the inner cell surface (lumen, S3/or in some literature referred as the tertiary layer) of a longitudinal tracheid (figure 1*b*). This type of cell composes above 90% of the volume of a softwood [11]. The morphology of a cut surface depends naturally on the cutting action. The cutting tool used, the speed and the cutting angle influence the structure of a created surface. Even if the surface can be cut perfectly, the microfibrils of a wood cell wall normally form aggregates (macrofibrils) in thickness of a few nanometres up to 60 nm [15], therefore a surface roughness with this minimum feature size is unavoidable. Furthermore, the inner surface of a cell entails natural texture, which is not altered by cutting. As a normal AFM tip has a radius of curvature which is comparable to the diameter of the macrofibrils, consideration of the wood surface roughness must be taken into account for the force measurement in this case. We examined the influence of substrate topography by selecting different locations of the same wood layer (S2) and comparing the results of adhesion measurements. We also discuss the influence of tip geometry by using tips with different sizes. Wood fibre consists of cellulose, hemicellulose, lignin and small amounts of other extractives, which differ in content at different locations [11]. On such a natural fibre surface, many factors influence the adhesion measured with AFM, including not only chemical composition, surface morphology, but also water absorption and cut surface oxidation/deactivation (fresh versus old surface). It is known that the freshly cut cell wall surfaces deactivate within the first 24 h and its polarity stabilizes afterwards [3]. Therefore, in order to achieve clear interpretation, it is necessary to limit the major variables that can influence the measurements of the adhesion forces. Time after surface cutting should be determined and a controlled environmental condition during measurements is desired.

AFM adhesion force measurements require that a tip approaches the surface from a distance, reaches repulsive force region, and then retracts so far from the surface until no surface forces are detected (electronic supplementary material, figure S1). In this measurement, different surface forces are acting on the tip, including attractive force, repulsive force and frictional force. We can roughly estimate the magnitude of pressure on the tip during a force measurement on a substrate as follows. According to the Hertz contact model where surface forces are ignored, the contact area can be calculated by equation (1.1):

1.1 $$a=\sqrt[{3}]{\frac{RF}{E_{\mathrm{tot}}}},\;\frac{1}{E_{\mathrm{tot}}}=\frac{3}{4}\left(\frac{1-\upsilon_{s}^{2}}{E_{s}}+\frac{1-\upsilon_{t}^{2}}{E_{t}}\right),$$

where *a* is the contact radius, *R* is the tip radius, *F* is the tip exerted on the surface, *E*_tot_ is the reduced Young's modulus, *E*_s_, *υ*_s_ and *E*_t_, *υ*_t_ are the Young's modulus and Poisson's ratio for sample and tip, respectively [1]. If the properties for a coniferous wood sample and Si tip are taken for these values: *F* = 1 nN, *R* = 10 nm, *E*_t_ = 150 GPa, contact modulus of the sample in the transverse direction $E_{s}/(1-\upsilon_{s}^{2})=10\;\text{GPa}$ [16], *υ*_s_ = 0.27, *υ*_t_ = 0.35, we obtain *a* = 0.9 nm. With given conditions, mean pressure would be 0.4 GPa. (*P* *=* *F/πa*^2^, with *πa*^2^ as the contact area.) It must be mentioned here that this is only a first estimation because the values of mechanical parameters vary considerably with respect to wood fibre orientation, moisture content, cellulose content, etc. Readers who are interested in the topic of anisotropic nano-indentation on wood can find details in the literature [16,17]. If large adhesive forces are considered, the pressure on an AFM tip can be even higher. Therefore, by multiple measurements, the wear of the tip must be considered. We analysed repeated measurements with commercially available tips, and demonstrated the influence of tip wear on the reliability of adhesion measurement.

## 2. Experimental section

The surface of a small cube (approx. 2 × 5 mm) of wood (spruce, *Picea abies*, three samples) was prepared with radial surface cut by a diamond knife (Diatome, DiS-Galetzka) mounted on a microtome (HM360 HistoServe, 1 mm s^−1^, knife angle 45°, cutting angle 5°). A detailed topography scan was carried out by non-contact mode AFM scan (less than 10 nm, 75 kHz tip). Each sample was stored in a dry atmosphere (less than 20% relative humidity (RH), 22°C ± 3°C) for one week before AFM adhesion force measurements to avoid the transient effect induced by ageing of a freshly cut surface. Force measurements were conducted in an environmental chamber where the sample was stored in an N_2_ filled atmosphere with 0% RH for at least 2 h. In this way, the influence of a surface water layer on the adhesion force could be minimized. Force measurements were conducted after a brief topography scan of the surface, so that the exact locations (either inner cell surface or cell wall) could be selected. Maximum deflection of 0.5 V is used for the repulsive force limit, which is less than the recommended contact mode deflection value (1 V) from the instrument (Agilent Technologies 5500 with multipurpose closed loop scanner) in order to reduce tip wear. The actual force depends on the deflection sensitivity (about 150 nm V^−1^, laser position dependent) and the force constant of the cantilever (0.07–0.4 N m^−1^, given by the supplier. Values calibrated with thermal noise method are shown in the electronic supplementary material). Contact mode cantilevers with common tip (NanoWorld Pointprobe® CONT probes, Si, standard shape, diameter less than or equal to 30 nm) and colloidal tip (sQube® colloidal probe, SiO_2_, spherical, diameter 2 µm) were used. The adhesion forces were measured within a 2 µm by 2 µm area for 64 locations (8 × 8 mapping), first at lumen (S3), afterwards at cell wall (S2). Then this procedure was repeated at the exact same locations three times. The adhesion forces measured within each 64 cycle were then plotted as a histogram and distribution characteristics were calculated. Tips after repeated measurement were examined by scanning electron microscope (SEM) and the AFM self-imaging technique with tip-check sample (figures are shown in the electronic supplementary material).

## 3. Results and discussion

### 3.1. Topography

**Figure 2.**
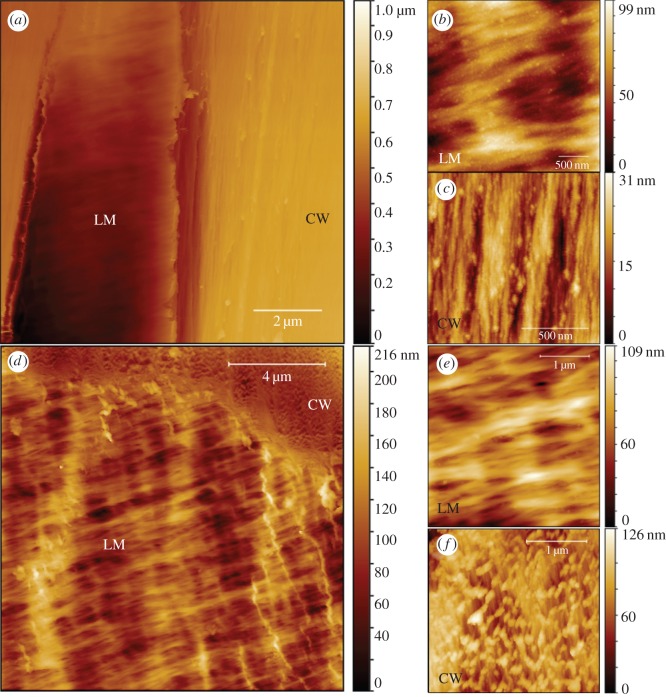
AFM topography image of a wood (spruce) radial surface prepared by microtome with a diamond knife at latewood (*a*–*c*) and earlywood (*d*–*f*) with magnified view of inner cell surface S3 (LM) in (*b*,*e*) and cell wall S2 (CW) in (*c*,*f*). As the local fibre orientation changes with respect to the cutting direction, the surface morphology of cell wall varies, as shown in (*c*) and (*f*).

Not only the cell type and location such as tracheid, S3/S2, earlywood/latewood, but also the local fibre orientation with respect to the cutting angle result in various morphology of the surface, as shown in figure 2. The macrofibrils of the inner cell surface S3 (lumen) form a characteristic wavy texture (figure 2*b,e*). The cut cell wall surface of earlywood (figure 2*d*,*f*) is generally rougher than the latewood region (figure 2*a*,*c*).

### 3.2. Roughness

The RMS roughness values were calculated from an AFM topography non-contact mode scan with a sharp tip (radius < 10 nm). The original AFM data were levelled by mean plane subtraction before roughness calculation, without further correction of tip dilation effect, and the same tip was used for different locations. RMS varies from 5 nm to above 30 nm per 4 µm^2^ area from position to position. For example, in figure 2*c* where the fibre direction is parallel to the cut surface, the roughness is lower than that in figure 2*f*, where the fibre direction is less parallel to the cut surface.

### 3.3. Adhesion force measurement by atomic force microscopy

**Figure 3.**
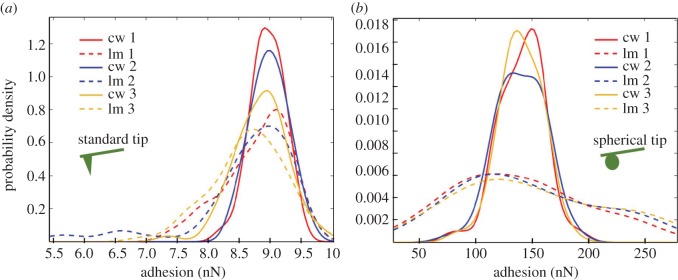
Distribution of adhesion forces. Each curve represents the non-parametric fitting of the distribution of adhesion forces from 64 measurements. Measurements were alternated between cell wall (cw) and lumen (lm) (sequence: lm-cw-lm-cw-lm-cw). Each change of location was repeated three times at the exact same locations using the identical tip. Two different tips, a standard Si tip with diameter less than or equal to 30 nm (*a*) and colloidal tip with diameter 2 µm (*b*) were used. The calibration information of the tips is shown in the electronic supplementary material.

Figure 3*a* shows the results of repeated measurements with a standard contact mode tip on a latewood location. Each curve represents the distribution of 64 adhesion forces measured within a 4 µm^2^ area at either cell wall (cw) or lumen (lm). Similar to a histogram, a kernel distribution fitting with normal distribution as smoothing function is applied to the measured adhesion forces and shown as probability density, in order to compare the differences between each set of measurements. It is visible in figure 3*a*, that the distribution gets wider after repeated measurements using the identical tip, both at cell wall and lumen. The peak value has the tendency to shift to lower values. The peak value of adhesion measured on lumen in the first run is on the right side of the peak value measured on cell wall in the first run (lm1 versus cw1); however, on the third run, it is shifted to the left side (lm3 versus cw3). It is clearly demonstrated that here measurement sequence plays a role in the result interpretation.

Two sources could be responsible for this observed effect here. Firstly, the sample surface ageing should be considered. Polymer surface directly after creation consists of dangling bonds and has generally higher surface energy. Such a fresh surface deactivates with time and therefore could result in changing of adhesion forces. As the sample was stored for one week after cutting before the adhesion measurements start, the time dependence of surface deactivation after cutting was minimized during these AFM measurements. Therefore, the major reason here is induced by the variance of the tip during measurements, i.e. tip wear. It has been reported, that a standard tip in pull-off force measurements commonly suffers from plastic deformation or even fracture [18]. Both SEM and AFM self-imaging confirmed the deformation of tips after repeated measurements (electronic supplementary material). Even though, the tip wear is a matter of individual handling and experienced users or different instrumental techniques can considerably reduce this effect, it is shown in our test, that the different values measured on lumen and cell wall can be compensated by the measurement sequence due to the existence of tip wear. Therefore, in order to gain reliable information by AFM adhesion measurement, it is necessary to prove that the tip wear does not have decisive influence on the results. This needs to be done by additional control tests or alternating measurements, which are not often shown in AFM force measurements in this field.

Another observation from figure 3*a* is that the distribution of adhesion forces on the lumen surface is wider than that on the cell wall surface measured by the same tip. This could result from a wider distribution in chemical composition, but also from greater surface roughness of the wood layers. From the discussion of surface topography before, it is clear that the surface roughness of natural fibre cannot be ignored. If the wide distribution of adhesion forces measured on lumen are owing to chemical composition, then the smaller the tip, the more distinction between chemicals at different locations will be shown, therefore the wider will be the distribution of adhesion on lumen. In order to examine the influence of tip size on the adhesion force measurement of wood, a colloidal tip, which has a spherical SiO_2_ particle (diameter 2 µm) attached to the cantilever was used. Measurements were carried out exactly at the same location on wood with the same parameters as in the experiment with smaller tips. The results are shown in figure 3*b*. It can be concluded from repeated measurements that the effect from tip wear is negligible for spherical tip and the distribution of adhesion forces is highly reproducible in this case. The seemingly unusual shape of cw2 could be caused by random experimental data and sensitive (not over-smoothened) curve fitting because the next repeated experiment (cw3) returns to normal shape (cw1). As the force constant of the cantilevers is similar, the force applied on the tips is also similar, but owing to the different size and curvature of the tips, the contact area, i.e. the pressure is largely different. Therefore, it is logical that the tip wear or modification is also different. It should also be mentioned here that the adhesion forces measured by the colloidal tip are very high and therefore effects such as nonlinearity of the photodiode would influence the accuracy of the absolute values of the calculated force.

In comparison with the results from the smaller tip (figure 3*a*), the difference between the distribution of adhesion forces measured on the lumen or cell wall is much larger in the case of a larger spherical tip. This observation supports the hypothesis that the difference in surface morphology (roughness and curvature) is the major influencing factor for the adhesion forces measured on cell wall and lumen. The curvature of lumen surface can increase contact area more significantly for a larger tip and hence induce more differences in the adhesion force measured on cell wall and lumen. However, this effect is negligible in these results because the chosen area for measurement is relatively flat compared to the rough texture according to the topography data (figure 2*b*).

**Figure 4.**
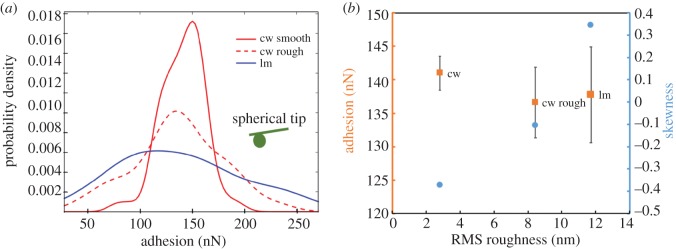
Roughness dependence of adhesion measured on several wood surfaces by AFM. (*a*) The histogram of 64 measurements conducted on a 4 µm^2^ area at two cell wall locations with different surface roughness and a lumen location. (*b*) The arithmetic mean of adhesion is plotted against the RMS roughness calculated from topography scan. The error bar shows the standard deviation (axis on the left). The skewness for the adhesion distribution is also plotted against RMS value (axis on the right).

To further analyse the influence of surface roughness, another location on the cell wall (S2) with higher surface roughness was selected. Results of adhesion measurements conducted on three different locations, a relatively smooth cell wall, relatively rough cell wall and naturally rough lumen are shown in figure 4. Locations of cell wall were limited to the S2 layer in order to minimize influencing variables. It is observed that the adhesion distribution gets wider as roughness increases. Comparing the results from the cell wall, increased roughness induces a positive shift of skewness. The result from lumen is exactly consistent with this observation, which also supports our hypothesis that the measured difference between lumen and cell wall with the current experimental set-up is induced by roughness. This point, to the best of our knowledge, has not been studied in previous works. Therefore, it is our opinion that future works related to this field should take the natural surface roughness into consideration.

## 4. Summary and conclusion

We have measured adhesion forces on wood surface with standard and colloidal tips by AFM under inert conditions. It is shown that the surface roughness of wood varies from location to location, which has a major influence on the adhesion distribution. Measurement sequence can play an important role in an AFM adhesion test on natural fibre surface, which should be considered for control experiments, such as repetition measurements with used tip, or an alternating measurement sequence on the areas of interest. Adhesion measured with an SiO_2_ colloidal tip is highly reproducible on the same location for a wood sample stored in inert conditions one week after surface cutting. Similar average value but different distributions are shown for the adhesion measured on cell wall and lumen. Evidences support the hypothesis that the major difference here is induced by surface roughness.

## Data accessibility

Supporting data are available at Dryad (http://dx.doi.org/10.5061/dryad.7g8cn) [19]. Software used in this study include: Matlab http://www.mathworks.com/products/matlab; Gwyddion http://gwyddion.net; Pico Image http://www.keysight.com
